# Supercritical CO_2_ extraction, chemical composition, and antioxidant effects of *Coreopsis tinctoria* Nutt. oleoresin

**DOI:** 10.1515/biol-2022-0092

**Published:** 2022-08-08

**Authors:** Yiyi Qiu, Hui Ruan

**Affiliations:** Department of Application Engineering, Zhejiang Institute of Economics and Trade, Xuelin Rd. 280, Hangzhou 310018, P. R. China; College of Biosystems Engineering and Food Science, Zhejiang University, Yuhangtang Rd. 866, Hangzhou 310058, P. R. China; Ningbo Innovation Center, Zhejiang University, Qianhunan Rd. 1, Ningbo 315100, P. R. China

**Keywords:** *Coreopsis tinctoria* Nutt., oleoresin, supercritical fluid extraction, response surface methodology, antioxidant activities

## Abstract

*Coreopsis tinctoria* Nutt. was used to extract oleoresin through supercritical CO_2_ extraction technology. The extraction conditions were optimized using response surface methodology, and the chemical composition of *C. tinctoria* Nutt. oleoresin (CTO) was analyzed. Under the optimal conditions, the antioxidant activity of oleoresin was determined using 1,1-diphenyl-2-picrylhydrazyl (DPPH˙) and 2,2′-azino-bis-(3-ethylbenzo-thiazoline-6-sulphonic acid)diammonium salt (ABTS˙^+^) free radical scavenging assays. The optimal extraction conditions were a 27.5 MPa extraction pressure, a 45°C extraction temperature, and a 3 h extraction time. Under these extraction conditions, oleoresin yield was up to 3.163%. Compared to steam distillation extraction, the CTO extracted using supercritical CO_2_ had more abundant components. The EC_50_ of CTO for DPPH˙ and ABTS˙^+^ free radical scavengers was 1.54 and 1.07 mg/mL, respectively.

## 1 Introduction

*Coreopsis tinctoria* Nutt., an annual herb widely distributed in North America, Central Asia, the Middle East, and Eastern Europe [1], grows in plateau areas in Xinjiang province, China. It is commonly known as Kunlun Snow Chrysanthemum because it grows all year in the snow-covered northern foothills of the Kunlun Mountains. Local Uyghurs refer to it as “Gulqai,” and it is used as a traditional medicine to prevent and treat hypertension and hyperlipidemia. Recent studies have reported that *C. tinctoria* has various biological activities, including hypoglycemic, hypotensive, anti-inflammatory, antioxidant, anticancer, antiaging, and antibacterial activities [2,3,4].

Oleoresin is a generic term for a kind of substance obtained from plants through oil dissolution, organic solvent extraction [5], three-phase partitioning [6], and supercritical fluid extraction (SFE) [7], which is composed of both volatile essential oils and nonvolatile compounds, such as pigments and fatty acids. Since oleoresin contains some natural antioxidants in the corresponding plants, it is more stable than essential oils [8]. According to literature reports, the supercritical extraction of oleoresin focused largely on spices like garlic, pepper (jalapeno) [9], and onion [10], among others. For spices, oleoresin better reflects flavor characteristics than essential oils obtained through hydrodistillation [11]. Furthermore, some studies have reported that oleoresin has stronger antioxidant and antimicrobial effects than essential oils [12,13,14]. While many studies are currently focusing on the flavonoids and polysaccharides of *C. tinctoria*, there are few studies devoted to *C. tinctoria* oleoresin (CTO), and challenges such as low extraction efficiency and poor application are common in research and development. Thus, it is important to optimize the extraction method and investigate its chemical composition for future studies.

The relative abundance of bioactive compounds in extracts from plant-derived sources depends on the extraction technique implemented [15]. Conventional organic solvent extraction, such as the Soxhlet extraction of oleoresins, has been widely adopted for its simplicity and economy. However, when compared to other extraction techniques, it is limited by its low efficiency and the large volume of solvents. Besides, its high operating temperatures are a major limitation, resulting in the formation of artifacts and the degradation of temperature-sensitive natural products [16].

Contrary to conventional extraction, supercritical CO_2_ extraction technology has a low extraction temperature, no toxic residue, and selective separation ability [17]. Therefore, it is particularly suitable for separating and extracting bioactive compounds with low concentration or low stability. The supercritical CO_2_ extraction technique was widely used for extra caffeine or decaffeination of tea at the industrial level [18,19], bringing high economic and medical value. Certainly, various researchers have produced plant oleoresin using supercritical CO_2_ extraction technology. Shukla et al. reported the supercritical CO_2_ extraction and online fractionation of dry ginger for the production of high-quality volatile oil and gingerol-enriched oleoresin, and the scale-up validation of this process has been achieved [20], revealing the potential of this technology. However, the insufficiencies caused by carbon dioxide emissions in this extraction process should be noted. It is a little contrary to the “low-carbon” concept. Thus, supercritical CO_2_ recycling is predicted to be a focus of studies.

In this study, the SFE of the CTO was optimized using the response surface methodology. Notably, we did not use co-solvents to accelerate extraction in this study, thereby substantially retaining the flavonoids and polysaccharides. The chemical composition of CTO was analyzed and compared with that of *C. tinctoria* essential oils (CTEOs). Furthermore, the antioxidant effects of CTO were determined through 1,1-diphenyl-2-picrylhydrazyl (DPPH˙) and 2,2′-azino-bis-(3-ethylbenzo-thiazoline-6-sulphonic acid)diammonium salt (ABTS˙^+^) free radical scavenging assays. We hope that this study provides basic data for high-value processing and utilization of *C. tinctoria*.

## 2 Materials and methods

### 2.1 Materials

*Coreopsis tinctoria* Nutt. (originated from Hetian, Xinjiang, batch number: 20190617-KLXJ) was purchased from Tongxiang Haitai Juye Co., Ltd. The DPPH kit was purchased from Sangon Biotech (Shanghai) Co., Ltd. The ABTS kit was bought from Beyotime Institute of Biotechnology, China. Dichloromethane and ethanol (analytical grade) were purchased from Sinopharm Chemical Reagent Co., Ltd, China.

### 2.2 Hydrodistillation extraction of CTEOs

The flowers of *C. tinctoria* Nutt. were dried in an oven at 60°C to a constant weight. Subsequently, they were hydrodistilled at a solid–liquid ratio of 1:10 for essential oil extraction.

### 2.3 SFE of CTO

The SFE was conducted on a laboratory-scale supercritical system (SFE-2; Applied Separations Inc., Pennsylvania, USA). The flowers of *C. tinctoria* Nutt. were dried in the oven at 60°C to a constant weight, and a certain amount of flower powder was pressed into the extraction vessel with degreasing cotton at both ends. The flow rate of carbon dioxide was fixed at 20 L/h. Different extraction pressures, temperatures, and times were chosen for single-factor experiments (Table 1). The oleoresin yield (*Y*) was calculated using the following equation:

(1) Y={m}_{1}/{m}_{2}\times 100 \% ,]

where *m*_1_ (g) is the weight of the extracted oleoresin and *m*_2_ (g) is the weight of the sample *C. tinctoria* used.

**Table 1:** Factors and levels in single factor experiment

Level	Time (h)	Pressure (MPa)	Temperature (°C)
1	0.5	15	35
2	1	20	40
3	1.5	25	45
4	2	30	50
5	2.5	35	55
6	3	—	—

### 2.4 Response surface methodology experimental design

Based on the results of the single-factor experiment, a three-variable Box–Behnken design with three coded levels was implemented to rapidly determine the optimal conditions for supercritical extraction. Scientific software Design-Expert (version 10.0.8.0; Stat-Ease Inc., Minneapolis, MN, USA) was employed for the experimental design. Three independent variables, extraction time (*X*_1_), extraction pressure (*X*_2_), and temperature (*X*_3_), were modified. The experimental range and the coded values of the three independent variables are listed in Table 2. The quadratic polynomial model proposed by the response surface methodology analysis for predicting the optimal combination can be expressed as the following equation:

(2) Y={\beta }_{0}+\sum {\beta }_{i}{X}_{i}+\sum {\beta }_{ii}{{X}_{i}}^{2}+\sum {\beta }_{ij}{X}_{i}{X}_{j},]

where *Y* is the yield of CTO; *β*_0_, *β*_*i*_, *β*_*ii*_, and *β*_*ij*_ are the regression coefficients for the intercept, linear, quadratic, and interaction terms, respectively; and *X*_*i*_ and *X*_*j*_ are the independent variables – time, temperature, and pressure. The significance of the model was evaluated, and the regression coefficient (*R* ^2^) was obtained.

**Table 2:** Factors and levers in the Box–Behnken experimental design

Level	*X*_1_ (time, h)	*X*_2_ (pressure, MPa)	*X*_3_ (temperature, °C)
−1	1	20	35
0	2	25	45
1	3	30	55

### 2.5 Chemical composition of CTEOs and CTO

GC-MS (7890B/7000C; Agilent Technologies, Palo Alto, PA, USA) was employed for the chemical composition analysis of CTO and CTEO, and an Agilent silica capillary column HP-5MS (30 m × 0.25 mm × 0.25 µm) was also used. For the chromatographic conditions, we followed the study by Wu et al. [21]. For the heating program, the initial temperature was set at 80°C and held for 3 min. Subsequently, the column was heated to 200°C at an 8°C/min rate and held for 5 min before being programmed to 260°C at an 8°C/min rate and held for 2 min. The sample solution was injected under a nitrogen atmosphere with a 10 mL/min flow rate. The sample dilution solution was prepared by dissolving a certain amount of CTO or CTEO in dichloromethane and then filtering it using a 0.22 µm membrane. An aliquot (1 µL) of sample dilution solution was injected under a helium carrier gas with a 1 mL/min flow rate, and the split ratio was fixed at 10:1. For the mass spectrometry conditions, the electron impact ion source energy was set as 70 eV, the ion source temperature was fixed at 250°C, and the mass scan range was *m*/*z* 30–600. Mass Hunter software (Agilent Technologies, Palo Alto, USA) was used for data analysis.

### 2.6 Antioxidant capability assay

#### 2.6.1 DPPH˙ free radical scavenging rate

The DPPH˙ free radical scavenging rate assay of CTO was conducted following Wu et al.’s [22] method, with minor modifications. CTO dissolved in dichloromethane was diluted to 0.15, 0.3, 0.6, 3, 6, 9, 12, and 18 mg/mL and mixed with equal volumes of DPPH˙ solution (0.1 mmol/L). The mixture was incubated in the dark for 30 min, and the absorbance at 519 nm was assayed. Dichloromethane was used as a control. Each measurement was conducted in triplicate. The DPPH˙ scavenging rates of the samples were calculated using the following equation:

(3) {\text{DPPH}}^{\text{˙}}\hspace{.25em}\text{scavenging rate}( \% )=({A}_{0}-{A}_{1})/{A}_{0}\times 100 \% ,]

where *A*_0_ and *A*_1_ represent the absorbance of the control and the sample, respectively.

#### 2.6.2 ABTS˙^+^ free radical scavenging rate

The ABTS˙^+^ free radical scavenging rate was determined using a rapid assay kit (Sangon Biotech, Shanghai, China) following Luo et al. [23]. First, the ABTS˙^+^ solution was mixed with an oxidant solution at equal volume to prepare the working stock solution. Second, the stock solution was incubated at 20–25°C in the dark for 12–16 h and then diluted with dichloromethane solution to 0.7 absorbance at 734 nm before use. Third, 10 µL of the diluted working solution was added to the sample solution to achieve final concentrations of 0.15, 0.3, 0.6, 3, 6, 9, 12, and 18 mg/mL and then incubated at 20–25°C for 4 min. Finally, the absorbance at 734 nm was determined. Each measurement was conducted in triplicate. The ABTS˙^+^ antioxidant effects can be calculated using the following equation:

(4) \begin{array}{c}{\text{ABTS}}^{ \textdotaccent +}\hspace{.25em}\text{free radical scavenging rate}\hspace{0.25em}( \% )\\ \hspace{1em}=({A}_{0}-{A}_{1})/{A}_{0}\times 100 \% ,\end{array}]

where *A*_0_ and *A*_1_ represent the absorbance of the control and the sample, respectively.

#### 2.6.3 EC_50_ estimation of antioxidant activity

EC_50_ estimation of antioxidant activity in DPPH˙ and ABTS˙^+^ assays using statistical programs based on Origin Pro 9.1 [24].

### 2.7 Statistical analysis

All experiments were implemented in triplicate. The differences between the mean values were analyzed by the Duncan test using SPSS Statistics 20 (IBM, Armonk, NY, USA). *P* < 0.05 was considered a statistically significant difference.

## 3 Results and discussion

### 3.1 Effects of variables on supercritical carbon dioxide extraction

**Figure 1:**
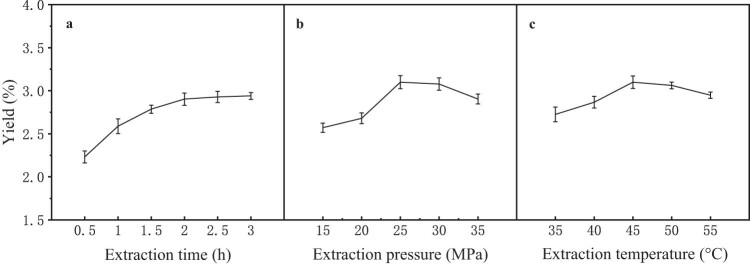
Effects of extraction time, extraction pressure, and extraction temperature on the yield of CTO.

The effects of each variable on the supercritical CO_2_ extraction of CTO were investigated using single-factor experiments. The results are shown in Figure 1. The effects of extraction time on the yield of CTO were studied under the following conditions: 45°C extraction temperature, 35 MPa extraction pressure, and 20 L/h CO_2_ flow rate. As shown in Figure 1a, the yield of CTO increased, but the extraction speed decreased with the extension of the extraction time. During the experiment, we also noticed that the color of CTO became darker as time increased. Although we set the experiment as a dynamic extraction, there was still some time before the temperature and the pressure were processed to the set value. This extended the extraction time and created a static extraction period; thus, the yield reached 50% of the total yield before 0.5 h. Barjaktarović et al. [25] studied the impact of time and pressure on the supercritical CO_2_ extracts of *Juniperus communis* L. fruits and proposed that 0–0.5 h is the “initial fast extraction period.” This is consistent with this study. Apart from the oleoresin yield, the extraction time can also affect the composition of oleoresins. Therefore, cost, efficiency, and component requirements should be considered when determining the extraction time. In this study, we chose 3 h for further investigation.

The impact of temperature is displayed in Figure 1c. Clearly, the yield reaches its maximum, and higher or lower temperatures result in lower yields. When the extraction temperature is lower than 45°C, a higher temperature means accelerated molecular thermal motion, increased coefficient of mass transfer, and greater volatility and diffusion speed, which induce a higher CTO yield. However, temperatures higher than 45°C will lower the solubility of CO_2_ by decreasing its density. Furthermore, excessively high temperatures will destroy CTO, which is highly volatile and heat sensitive. Thus, 45^o^C was selected as the center point in the response surface methodology.

After the time and temperature of the center point had been chosen, the effect of extraction pressure was investigated. The pressure was fixed at 10, 15, 20, 25, and 30 MPa when the time, temperature, and CO_2_ flow rate were set at 3 h, 45°C, and 20 L/h. The results are shown in Figure 1b. When the extraction pressure was between 10 and 25 MPa, a higher pressure would increase the density of supercritical CO_2_, which has greater solubility for oleoresins. In addition, high pressure acting on materials in the vessel may accelerate the dissolution of the CTO. When pressure is higher than 25 MPa, the yield of CTO shows a downward trend. This may be due to the decrease in CO_2_ fluid mobility caused by high pressure over a certain value [26]. In addition, the color of the CTO becomes darker with the increased pressure, indicating that the pressure also influences the components of the CTO. Gaspar [27] discovered that as the pressure increases, more phytowax would be extracted and cause the deterioration of plant oil. Consequently, we chose 20, 25, and 30 MPa for the response surface methodology.

### 3.2 Optimization of CTO SFE using response surface methodology

A three-level Box–Behnken design with three variances was constructed, setting extraction time (*X*_1_), extraction temperature (*X*_2_), and extraction pressure (*X*_3_) as the variances and the CTO yield as the response value (*Y*), leading to 17 sets of experiments with five replicates at the center point. The results are listed in Table 3. We used Design-Expert to process the data by polynomial regression and generated the following polynomial quadratic equation:

(5) \begin{array}{c}Y=3.11+0.21{X}_{1}+0.26{X}_{2}+0.15{X}_{3}-0.025{X}_{1}{X}_{2}\\ \hspace{1em}-0.027{X}_{1}{X}_{3}-0.15{X}_{2}{X}_{3}-0.2{{X}_{1}}^{2}-0.31{{X}_{2}}^{2}-0.25{{X}_{3}}^{2}.\end{array}]

**Table 3:** Design and its results for the surface methodology experiment

Runs	*X*_1_	*X*_2_	*X*_3_	*Y* (yield, %)
1	0	1	1	2.81
2	0	−1	−1	1.97
3	0	1	−1	2.74
4	0	−1	1	2.64
5	1	0	1	2.99
6	−1	0	−1	2.27
7	1	0	−1	2.80
8	−1	0	1	2.57
9	1	1	0	3.04
10	−1	−1	0	2.10
11	1	−1	0	2.53
12	−1	1	0	2.71
13	0	0	0	3.03
14	0	0	0	3.07
15	0	0	0	3.13
16	0	0	0	3.14
17	0	0	0	3.16

**Figure 2:**
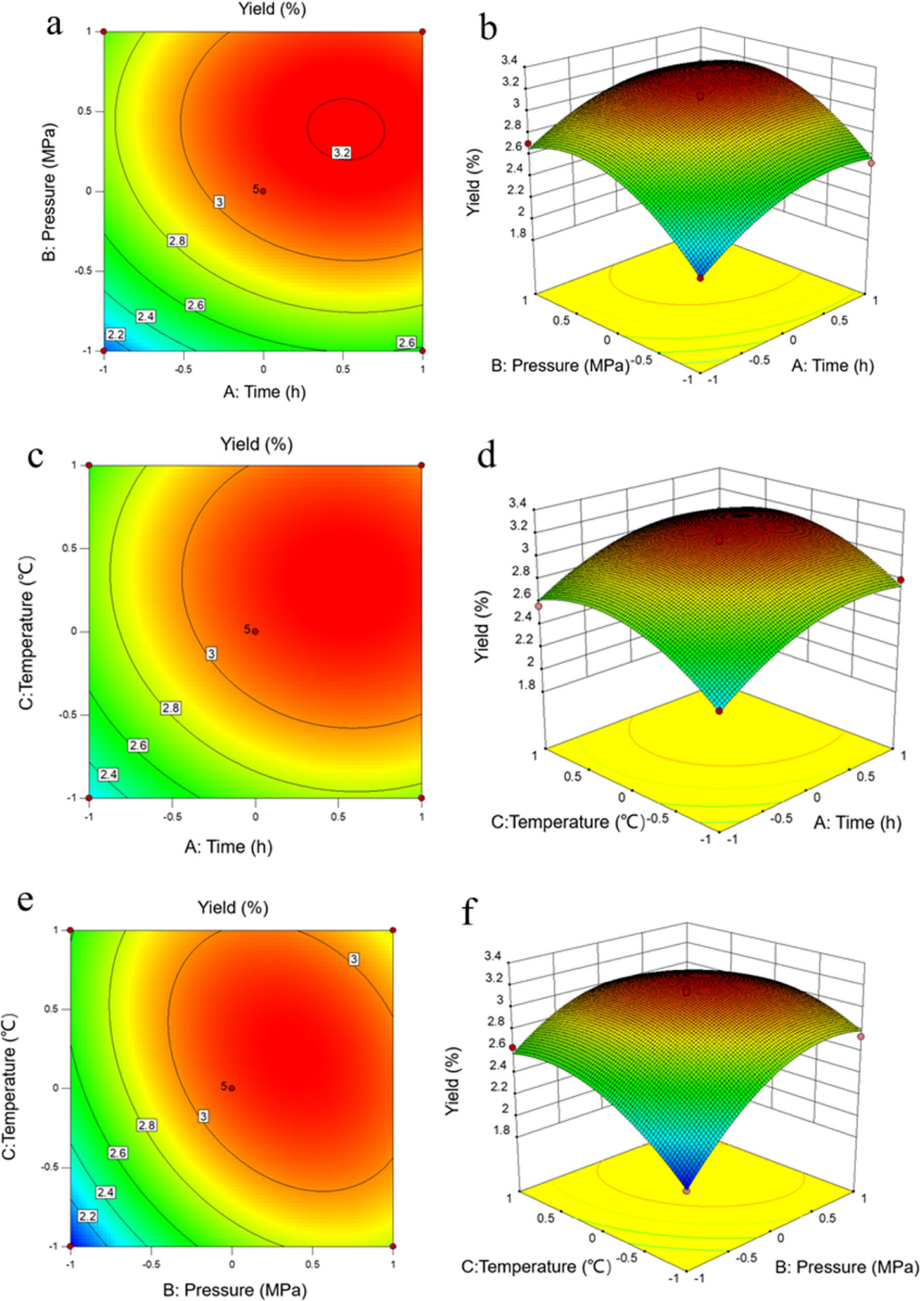
Response surface and contour line for predicted equation: (a and b) time and pressure (c and d) time and temperature, and (e and f) pressure and temperature.

Table 4 demonstrates the results of the analysis of variance. The *p*-value of this model is lower than 0.0001, indicating that the polynomial quadratic equation constructed by this model is extremely significant. The coefficient of determination (*R* ^2^) is 0.9869, which indicates a good fit, and 98.69% of the variability in the response originates from the selected variances [28]. The adjusted coefficient of determination (*R* ^2^_adj_) is 0.9701, suggesting that 2.99% of the total variation could not be explained by the model. The lack of fit is not significant at *α* = 0.05, indicating that the residual is caused by errors, and the model can be used for result prediction. The visualization of the generated equation is displayed by response surfaces in Figure 2.

**Table 4:** Results of the response surface methodology regression analysis for CTO yield

Source	Sum of squares	df	Mean square	*F*-value	*p-*value	Significance
Model	2.12	9	0.24	58.71	<0.0001	Significant
*X*_1_	0.37	1	0.37	91.07	<0.0001	***
*X*_2_	0.53	1	0.53	132.16	<0.0001	***
*X*_3_	0.19	1	0.19	47.12	0.0002	***
*X*_1_ *X*_2_	2.500 × 10^−3^	1	2.500 × 10^−3^	0.62	0.4559	
*X*_1_ *X*_3_	3.025 × 10^−3^	1	3.025 × 10^−3^	0.75	0.4141	
*X*_2_ *X*_3_	0.090	1	0.090	22.42	0.0021	**
*X*_1_^2^	0.16	1	0.16	40.61	0.0004	**
*X*_2_^2^	0.42	1	0.42	103.60	<0.0001	***
*X*_3_^2^	0.27	1	0.27	66.49	<0.0001	***
Residual	0.028	7	4.014 × 10^−3^			
Lack of fit	0.016	3	5.458 × 10^−3^	1.86	0.2766	Not significant
Pure error	0.012	4	2.930 × 10^−3^			
Cor total	2.15	16				
*R* ^2^	0.9869					
*R* ^2^_Adj_	0.9701					

Notes: ***extremely significant (*p* < 0.0001); **highly significant (*p* < 0.01); *significant (*p* < 0.05).

In the model, *X*_1_, *X*_2_, and *X*_3_ are all extremely significant, and the order ranked according to their *p*-value is as follows: *X*_2_ (extraction pressure) > *X*_1_ (extraction time) > *X*_3_ (extraction temperature). The influence of variances can also be interpreted by the response surface and the contour plots. Each response surface and its corresponding contour plots represent the effect and mutual interaction of two variances against the response. From Figure 2a and b, with the increase of time or pressure, the yield of CTO first increased and then diminished. The change resulting from a pressure boost is more powerful than that of the time, suggesting that the extraction pressure has a more significant impact on the CTO yield. Figure 2c and d illustrates the effects of time and temperature. Clearly, time is slightly stronger than temperature. Figure 2e and f advocates that the pressure is a much more effective variance than the temperature. A high temperature would lead to a greater Brownian motion, beneficial to oleoresin extraction. Similarly, high pressure will enhance the solvation effect of CO_2_ fluid and further boost the CTO yield. However, excessively high temperature and pressure would hinder the dissolution of the CTO.

For the quadratic model generated by the response surface methodology, the first derivate of the equation can be applied to access the optimal conditions [29]. In this study, we employed Design-Expert for the calculation. The optimal condition was as follows: 27.5 MPa extraction pressure, 45.69°C extraction temperature, and 3 h extraction time. The predicted highest CTO yield was 3.163%. Considering the limitations in practice, we fixed the conditions at 27.5 MPa extraction pressure, 46°C extraction temperature, and 3 h extraction time. Under these conditions, we performed the extraction in triplicate, and the average value was 3.165%, which is consistent with the predicted value. In conclusion, the conditions optimized by the response surface methodology have good reliability and application value.

Liu et al. [30] extracted CTO under 60°C, 25 MPa, and 45 L/h CO_2_ flow rate for 1 h, and the yield was 11. 59% ± 0. 37%. The absolute value of the olefin content in the report was similar to that of this study, implying that Liu et al. might obtain more vegetable wax under a higher temperature. In addition, high temperature has negative effects on the quality of olefins. Thus, the CTO extracted by the present study may have higher olefin content and more potent antioxidant or antibacterial activities.

### 3.3 Chemical composition comparison between CTO and CTEOs

GC-MS was implemented for the chemical composition analysis of CTO and CTEO. Data were processed by the normalization method in Mass Hunter loaded with the NIST 11.L library. The main components detected by the GC-MS of CTO and CTEO are shown in Table 5. CTO and CTEO have a significant difference in their chemical compositions – 49 compounds were detected in CTO, whereas only 32 compounds were distinguished in CTEO. In addition, SEF extraction obtained more alkenes than steam distillation extraction (calculated by the alkenes yield per unit material mass). However, CTEO has more kinds of alkenes than CTO. Those alkenes not detected in CTO are also relatively low in CTEO; hence, they may exist in CTO but become undetectable by GC–MS.

**Table 5:** Main chemical components of *Coreopsis tinctoria* Nutt. extracted by SFE

No.	Compound	CTO (%)	CTEO (%)
1	γ-Terpinene		7.47
2	Camphene		0.13
3	3-Carene		0.16
4	(+)-3-Carene		1.11
5	*o*-Cymene	1.88	16.54
6	*p*-Cymene		0.43
7	d-Limonene	8.21	40.69
8	Acetophenone	0.86	
9	γ-Terpinene		0.29
10	Carveol	2.55	5.71
11	(−)-Carvone	1.15	3.23
12	2,4,6-Trimethylbenzyl alcohol		0.45
13	Tricyclo[5.2.1.0(1,5)]decane	0.17	
14	Cyclohexane, 2-ethenyl-1,1-dimethyl-3-methylene-		0.13
15	Bicyclo[3.1.0]hexan-3-ol, 4-methylene-1-(1-methylethyl)-, [1*S*-(1α,3β,5α)]-	0.69	
16	1,2-Dimethyl-		1.24
17	α-Campholenal		0.46
18	1,3,8-*p*-Menthatriene		1.17
19	4-Terpinenyl acetate		0.45
20	2,4-Dimethylstyrene		0.44
21	β-Humulene	0.18	
22	1,2-Cyclohexanediol, 1-methyl-4-(1-methylethenyl)-	2.35	
23	Epiglobulol	0.14	
24	Lanceol	0.18	
25	(−)-β-Chamigrene	0.12	
26	Di-epi-α-cedrene	0.26	
27	γ-Himachalene	0.19	3.75
28	Caryophyllene		0.21
29	*δ*-Selinene		0.21
30	*Cis*-sesquisabinene hydrate	0.14	
31	Ascaridole epoxide	0.18	
32	1,5,5-Trimethyl-6-methylene-cyclohexene		0.25
33	1,4-Cyclohexadiene, 3-ethenyl-1,2-dimethyl-		1.24
34	Thymol		0.28
35	Bicyclo[5.2.0]nonane, 2-methylene-4,8,8-trimethyl-4-vinyl-	0.15	
36	Bicyclo[3.1.1]hept-2-ene, 2,6-dimethyl-6-(4-methyl-3pentenyl)	2.39	
37	Naphthalene		0.16
38	3-Cyclohexene-1-acetaldehyde, α,4-dimethyl-	0.33	
39	Benzene, 1-(1,5-dimethyl-4-hexenyl)-4-methyl	0.31	0.69
40	γ-Muurolene	1.64	
41	Dodecanoic acid	0.12	
42	*Trans*-*Z*-α-bisabolene epoxide	0.06	
43	*Trans*-longipinocarveol	0.10	
44	(−)-Spathulenol	0.27	
45	Alloaromadendrene oxide	0.59	
46	Aromadendrene oxide	0.08	
47	Aromadendrene, dehydro-		0.18
48	Isoaromadendrene epoxide	0.07	
49	Tricyclo[6.3.0.0(1,5)]undec-2en-4-one, 2,3,5,9-tetramethyl-	0.58	
50	Fluorene	3.89	4.95
51	*Trans*-longipinocarveol	0.22	
52	Ledene oxide-(II)	0.15	
53	Berkheyaradulene		0.24
59	Phytol, acetate	0.45	
60	9,12-Octadecadienoic acid (*Z*, *Z*)	4.01	
61	9,12,15-Octadecatrienoic acid, (*Z*, *Z*, *Z*)	2.04	
62	Eicosane	1.88	
63	Behenic alcohol	5.03	
64	Heneicosane	5.94	0.72
65	1-Dodecanol, 2-octyl	16.21	
66	Squalene	1.88	
67	Acetic acid, chloro-, octadecyl ester	2.15	
68	Pentacosane	14.07	
69	Tetradecane, 2,6,10-trimethyl	1.59	
70	Tetracosane	5.28	
71	Phytol	0.94	0.24
Total		91.67	93.22

2-Octyl-1-dodecanol, pentacosane, and d-limonene are the three most abundant compounds in CTO, accounting for 16.21, 14.07, and 8.21% of the total peak area. 2-Octyl-1-dodecanol and pentacosane are waxes of plant cuticles [31]. 2-Octyl-1-dodecanol can be used as an emulsifier, solvent, and thickening agent, indicating the further exploitation of *C. tinctoria*. Pentacosane is a kind of higher aliphatic hydrocarbon that is common in plant SFE extract. d-Limonene is the highest compound in CTO alkenes, accounting for 18.15% of the total peak area. It is a monoterpene with several biological activities, including antioxidant, anticancer, asthmatic, anti-inflammatory, and antimicrobial effects [32], and has been widely applied as a food preservative [33]. γ-Muurolene and *o*-cymene are also important contents in CTO, accounting for 1.88 and 1.64%, respectively. γ-Muurolene, a sesquiterpene that is rich in *Cananga odorata* (Lam.) Hook.f. & Thomson and birch bud essential oils, is famous for its special scent. *o*-Cymene and its isomer, *p*-cymene, are derivatives of α-pinene and play a synergistic role in the antibacterial process of *Thymus mongolicus* Ronn essential oils [34]. Free fatty acids and fatty acid esters are two other kinds of important compounds in CTO, accounting for 7.76% of the total peak area, including linoleic acid, linolenic acid, and lauric acid. Reportedly, these free fatty acids are bioactive compounds. For instance, linoleic acid has a potent antibacterial effect against *Mycobacterium tuberculosis* in the MGIT 960 system, and its minimum inhibitory concentration is 200 μg/mL [35]. It is noteworthy that acetic acid, chloro-, octadecyl ester is unlikely to be found naturally in *C. tinctoria* Nutt. This may be due to environmental contamination or a mismatch in the libraries. Further study is needed to confirm the origin of this substance.

### 3.4 Antioxidant activities

**Figure 3:**
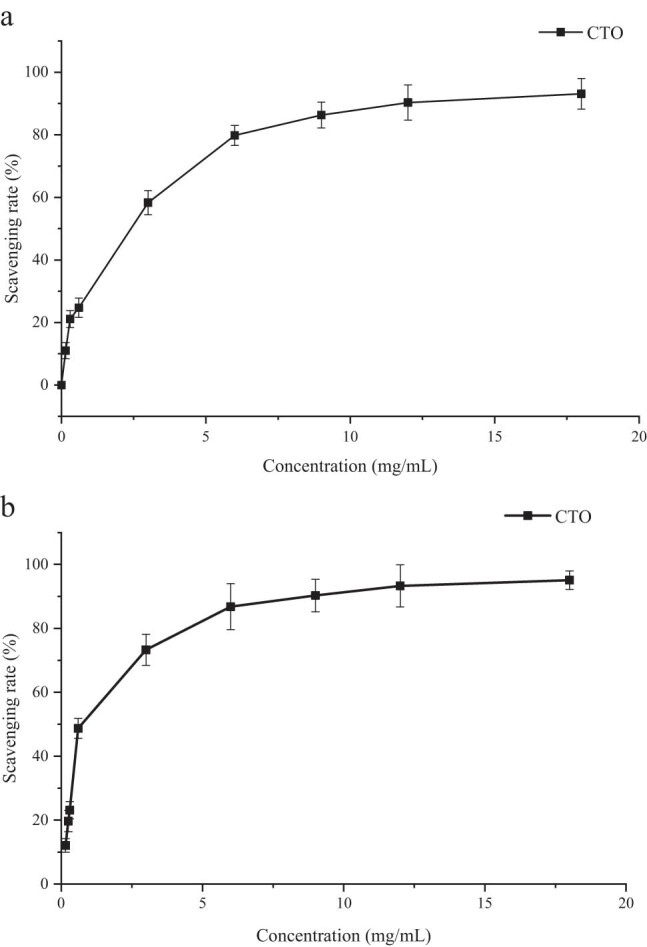
DPPH˙ free radical scavenging rate (a) and ABTS˙^+^ free radical scavenging rate (b) of CTO.

The DPPH˙ and ABTS˙^+^ free radical scavenging capabilities of CTO were assayed, and Trolox was used as the positive control. The results are displayed in Figure 3. Evidently, CTO has a strong free radical scavenging capacity, and its EC_50_ against DPPH˙ and ABTS˙^+^ is 1.54 and 1.07 mg/mL, respectively. In earlier reports, SFE extracts always exhibit high antioxidant effects due to their mild extraction conditions and protection by supercritical CO_2_ [36,37]. Teixeira et al. [38] tested 17 kinds of commercial essential oils and found that while seven essential oils had EC_50_ ranging from 0.04 mg/mL to 10.4 mg/mL, others had almost no antioxidant capacity. In contrast, CTO has a strong antioxidant capacity. However, several studies have reported that oleoresins from different plants have a much more potent DPPH˙ free radical scavenging ability than the CTO extracted in this study [5,39,40], which may be due to the differences in extraction methods. The solvent extraction they used could extract many nonvolatile antioxidant compounds, such as flavones, leading to high free radical scavenging rates. The relatively low scavenging rate of CTO suggests that the SFE conditions optimized in this study did not extract flavones and other similar compounds. Thus, the flowers that have been extracted by SFE to obtain oleoresin can be used for further flavonoid extraction.

## 4 Conclusion

In conclusion, this study constructed a CTO SFE extraction method with conditions optimized using response surface methodology. The optimal conditions were 27.5 MPa as the extraction pressure, 45.69°C as the extraction temperature, and 3 h as the extraction time. The maximum CTO yield was 3.163%. The chemical composition of CTO was analyzed and compared with that of CTEO. Furthermore, the antioxidant effects of CTO were determined through DPPH˙ and ABTS˙^+^ free radical scavenging assays. CTO had more abundant components than CTEO and had higher alkene content. The antioxidant activities of CTO were excellent, and its EC_50_ against DPPH˙ and ABTS˙^+^ was 1.54 and 1.07 mg/mL, respectively. We hope this study provides theoretical guidance for CTO extraction and new insights into the further exploitation and application of *C. tinctoria*.

## Figures and Tables

**Figure A1:**
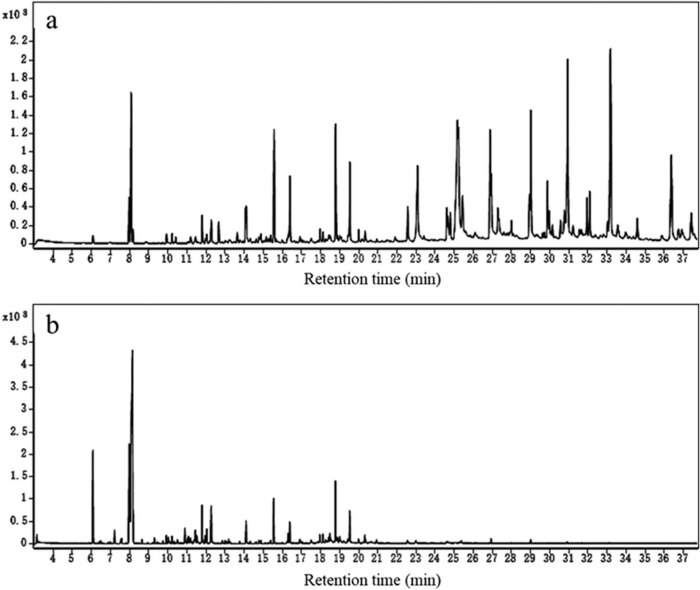
GC-MS chromatograms of CTO (a) and CTEO (b).
